# BORIS: a key regulator of cancer stemness

**DOI:** 10.1186/s12935-018-0650-8

**Published:** 2018-10-05

**Authors:** Sara Soltanian, Hesam Dehghani

**Affiliations:** 1 Department of Biology, Faculty of Sciences, Shahid Bahonar University of Kerman, Kerman, Iran; 2 Department of Basic Sciences, Faculty of Veterinary Medicine, Ferdowsi University of Mashhad, Azadi Square, Mashhad, 91775-1793 Iran; 3 Division of Biotechnology, Faculty of Veterinary Medicine, Ferdowsi University of Mashhad, Mashhad, Iran; 4 Stem Cells and Regenerative Medicine Research Group, Research Institute of Biotechnology, Ferdowsi University of Mashhad, Mashhad, Iran

**Keywords:** BORIS, Cancer, Gene expression, Cancer stem cell, Pluripotency, Epigenetic modification

## Abstract

BORIS (CTCFL) is a DNA binding protein which is involved in tumorigenesis. Although, there are different opinions on the level of gene expression and function of BORIS in normal and cancer tissues, the results of many studies have classified BORIS as a protein belonging to cancer/testis (CT) genes, which are identified as a group of genes that are expressed normally in testis, and abnormally in various types of cancers. In testis, BORIS induces the expression of some male germ cell/testis specific genes, and plays crucial roles during spermatogenesis and production of sperm. In tumorigenesis, the role of BORIS in the expression induction of some CT genes and oncogenes, as well as increasing proliferation/viability of cancer cells has been demonstrated in many researches. In addition to cancer cells, some believe that BORIS is also expressed in normal conditions and plays a universal function in cell division and regulation of genes. The following is a comprehensive review on contradictory views on the expression pattern and biological function of BORIS in normal, as well as cancer cells/tissues, and presents some evidence that support the expression of BORIS in cancer stem cells (CSCs) and advanced stage/poorer differentiation grade of cancers. Boris is involved in the regulation of CSC cellular and molecular features such as self-renewal, chemo-resistance, tumorigenicity, sphere-forming ability, and migration capacity. Finally, the role of BORIS in regulating two important signaling pathways including Wnt/β-catenin and Notch in CSCs, and its ability in recruiting transcription factors or chromatin-remodeling proteins to induce tumorigenesis is discussed.

## Background

Brother Of the Regulator of Imprinted Sites (BORIS) or CTCFL (CCCTC-binding factor like) protein is recognized as a paralog of CTCF (CCCTC-binding factor). CTCF is a DNA binding protein that is involved in chromatin insulation, genomic imprinting, intra/interchromosomal interactions, and global three-dimensional genome organization [1–6]. BORIS and CTCF have identical 11 Zinc finger DNA-binding domains, and both seem to bind to similar DNA target sequences [7]. However, a study by Pugacheva et al. showed that only a subset of CTCF binding regions in cancer is occupied by BORIS [7, 8]. In spite of the very similar DNA binding domain in these two proteins, their amino and carboxyl domains have very little sequence homology, leading them to interact with different partners. Therefore, it may be the protein partners of these two proteins that determine their different chromatin regulating abilities and functional outcomes [1, 9–12]. The human *BORIS* gene is located at 20q13 and is comprised of 11 exons, 10 of which are coding [1]. Pugacheva el al. characterized 23 transcript variants of BORIS resulting in 17 protein isoforms. Different isoforms contain different zinc-fingers in their DNA-binding domain, have different amino and carboxyl termini, and have distinct expression profiles in various normal and cancer cells [13].

Many studies have attempted to explain the roles of BORIS in different cell types. Problems in understanding the biological roles of BORIS can be attributed to the lack of knowledge about the expression patterns of its isoforms in diverse cell types, the unknown identity of its potential interacting partners, and the experimental, analytical, and biological variability of the experiments performed [14]. According to many reports, BORIS is generally classified as a member of cancer testis (CT) genes, a group of genes which are normally expressed in germ cells, notably in testis, and also in a wide range of cancer types [15–18]. High expression of BORIS in testis suggests its involvement in the regulation of specific testis genes and meiosis of sperm [7–9, 19–22]. Abnormal expression of BORIS in a variety of cancer cells/tissues has been the main reason to categorize it as an oncogene with pathogenic roles in cell proliferation and tumorigenesis [7, 11, 13, 15–18, 21, 23–38]. Specific expression of BORIS in cancer stem cell (CSC) population and its role in the induction and maintenance of some important CSC properties suggest an association with severe malignancy and advanced stages of cancer [14, 32, 34, 39–50]. Several researchers reinforce the view that the expression of BORIS might not be limited to cancer cells/tissues and it might also be expressed in normal tissues and cells, and have a universal function [16, 17, 25, 27, 30, 51–53].

In this review, we explain in detail the reports that are related to the expression and general function of BORIS in normal tissues/cells such as testis/male germ cells. Subsequently, the expression of BORIS in various cancer/cancer stem cells, and its role in cell proliferation, tumorigenesis, and maintenance of CSC properties will be discussed. Finally, a mechanism for BORIS-mediated function in cancer and CSCs to regulate the expression of target genes and to induce tumorigenesis will be discussed.

## Expression pattern and role of BORIS in normal cells/tissues

The first reports demonstrated that in contrast to the ubiquitous expression of CTCF in all somatic cell types, BORIS expression is restricted to testis. They also showed that during male germ cell development, BORIS and CTCF are expressed in a mutually exclusive manner. While CTCF expression was detected in post-meiotic round spermatids and spermatozoa, the expression of BORIS was only detected in primary spermatocytes, a cell type without CTCF expression. This finding indicated that the activation of BORIS expression is linked with the final round of mitosis of male germ-line cells [1, 18]. However, in subsequent studies, it was shown that BORIS is also expressed in pre-meiotic spermatogonia and pre-leptotene spermatocytes, where the expression of CTCF was also detected [21].

Thus far, some functions have been attributed to BORIS in testis. In fact, an extensive overlap has been recorded between the genome-wide erasure of methylation, re-setting of paternal DNA methylation patterns, and BORIS expression/silencing of CTCF [18], indicating that in testis, BORIS may play a role in the reprogramming of the paternal DNA [4, 18]. BORIS has also been implicated to be involved in the resetting of imprinting at the Igf2/H19 imprinting control region (ICR) in male germ cells [10]. In contrast, in somatic cells, CTCF is recognized as reader and protector of Igf2/H19 imprinting marks [11–13, 18, 21]. In addition, during spermatogenesis, BORIS has been detected as an inducer of multiple testis-specific genes which are suppressed by CTCF in somatic cells [7–9]. For example, important roles of BORIS in the induction of expression of some male germ cell/testis specific genes including ALF, SPANX-N, Gal3st1, and Prss50 which play crucial roles in meiosis and spermatogenesis have been reported [19–22]. This is consistent with the findings in BORIS knockout male mice which show subfertility and multiple defects in spermatogenesis, including a reduction in testis size, defective sperm production and a significant delay in the production of sperm [21, 22]. Overall, these studies show that in testis tissue, BORIS regulates gene expression, and exerts an important role in meiosis and production of the haploid sperm.

Although according to some reports, repressive effects of CTCF, p53, and promoter DNA methylation has restricted the expression of BORIS to testicular germ cells [1, 7, 11, 13, 15, 24, 25, 28], a few other studies have shown that in addition to male germ cells, BORIS transcripts are also expressed in other normal tissues such as human oocyte and ovary, and in various fetal tissues [13], indicating a role in meiosis during oogenesis [13, 44, 54], and early stages of preimplantation development [44] (Table 1). Significant levels of BORIS were also found in normal human skin and freshly isolated whole dermis, epidermis, or disaggregated primary keratinocytes [52] (Table 1).

**Table 1:** Cells/tissues that normally express BORIS

Normal cell line or tissues	mRNA/protein level	References
Male germ cells, human and mouse testis	mRNA and protein	[1, 18, 51]
Oocyte	mRNA	[44]
Primary keratinocytes	mRNA and protein	[52]
Mouse fibroblast cell line (STO-3T3)	mRNA	[56]
Human lung fibroblasts cell line (MRC5)	mRNA and protein	[51]
Human ovary	mRNA	[13, 51, 54]
Human skin	mRNA and protein	[13, 52]
Human prostate and bladder tissues	mRNA and protein	[30, 51]
Human adipose, brain, cervix, colon, esophagus, kidney, liver, placenta, muscle, spleen, thymus, thyroid, trachea	mRNA and protein	[51]
Mouse cerebellum, gut, kidney, liver, ovary, spleen	mRNA and protein	[51]

Several general regulatory functions have been proposed for BORIS in normal cells. Rosa-Garrido et al. exhibited that BORIS is involved in RNA transcription, cell cycle progression, and genome instability [52]. Experiments using the ectopic expression or inactivation of BORIS demonstrated that optimal levels of BORIS is needed to support normal cell division. In addition, BORIS knock-down caused a reduction in the synthesis of rRNA and global RNA, suggesting a role for BORIS in the licensing of RNA transcription [52]. BORIS has also been recognized as a RNA binding protein which is associated with actively translating ribosomes. These properties display its role in the regulation of genes at both the transcriptional and post-transcriptional levels [55]. Moreover, localization of BORIS within the nucleolus of cancer and normal cells suggested a role for this protein in nucleolar function [51].

## Expression pattern of BORIS in cancer cells/tissues

In many tumors and cancer cell lines, hypomethylation of BORIS promoter leads to overexpression of BORIS [11, 13, 15–17, 23–33]. For instance, Vatolin et al. and Hong et al. demonstrated that the suppressed expression of BORIS (observed in normal somatic tissues and cell lines), is abrogated in various breast, neuroblastoma, prostate, melanoma, colon, and lung cancers [11]. In other studies, the comparative expression analysis of several cancer/testis genes revealed a high incidence of BORIS expression in uterine/endometrial, ovarian and cervical cancers in comparison with their normal tissues [17, 24, 32, 56]. In similar reports, analysis of BORIS in esophageal squamous cancers, pancreatic and hepatocellular carcinoma indicated that the expression of BORIS was significantly higher in these cancers than that in the adjacent non-cancerous tissues and normal cells [46, 47, 49, 57]. Some reports on prostate cancer, glioblastoma, and laryngeal squamous cell carcinoma indicate that BORIS protein is absent or present at low levels in non-tumorigenic cells and tissues, but it is present at variable higher levels in all cancer cell lines and tumors, indicating that BORIS might be used as a cancer biomarker [48, 50, 58]. D’Arcy and colleagues also showed that BORIS is expressed in all types of breast cancer cell lines, whereas primary normal breast cells and normal breast tissues do not express this protein [15]. The other evidence in support of BORIS as a tumor marker was obtained by detecting significantly higher level of BORIS in the leukocyte fraction in patients with different types of breast tumors compared to the control group [23]. Although, there is no report about BORIS expression in leukemic patients, some isoforms of BORIS are detected at high levels in leukemic cell lines [13]. Overall, the expression of BORIS in testis and many cancers (Table 2) led to its classification as a CT gene [15–18].

**Table 2:** Expression of BORIS in different cancer cells/tissues

Cancer cell line or tumor tissue	mRNA/protein level	References
Neuroblastoma	mRNA	[60]
Breast cancer	mRNA and protein	[15, 60]
Leukemic cell lines	mRNA	[13]
Ovarian cancer	mRNA	[17]
Colon cancer	mRNA	[60]
Prostate cancer	mRNA and protein	[48, 60]
Uterine cancer	mRNA	[24]
Cervical cancer	mRNA	[32]
Endometrial cancer	mRNA	[57]
Esophageal squamous cancer	mRNA and protein	[49]
Pancreatic cancer	Protein	[58]
Hepatocellular carcinoma	mRNA and protein	[31, 47]
Glioblastoma	mRNA and protein	[59]
Laryngeal squamous carcinoma	mRNA and protein	[50]
Melanoma	mRNA	[60]

In contrast to numerous reports indicating the expression of BORIS in cancers, some researchers report different findings. For instance, although BORIS is activated in a substantial fraction of melanoma samples, it does not appear to be present in all tumors of this kind [16]. Another research indicated that immortalized human ovarian surface epithelial cells (IOSE121) and four ovarian cancer cell lines (OVCAR3, SKOV3, A2780, and OVCAR429) do not express BORIS or other CT genes at significantly higher levels [17]. In a research by Hines et al., it was revealed that neither mature BORIS transcripts nor spliced variants are commonly expressed at detectable levels in human breast cancer cell lines and high grade breast carcinomas. There are also reports that show the absence of a significant difference in BORIS transcript levels in cancer and non-cancer cells. For example, expression of BORIS mRNA showed no significant difference between normal and cancerous prostate and bladder tissues [30], and also between some mouse cancer and non-cancer cell lines [59]. Similarly, according to the findings of Sheer and colleagues, the expression of BORIS was not restricted to the germ/cancer cells and its expression was also detected within the nucleolus of normal and cancer cells [51, 53].

Therefore, the above findings demonstrate the widespread expression of BORIS in normal and cancer cells. In reality, one reason for detection of BORIS in a variety of cell lines can be related to loss of the q arm of chromosome 16 (the locus of CTCF as a suppressor of BORIS) and gain of chromosome 20q13 (the locus of BORIS) during prolonged growth of normal and cancer cell lines in culture, a phenomenon that occur throughout adaptation of hES cells to growth in culture for a long time [60, 61]. Moreover, the use of different techniques to measure the expression of BORIS, isolated detection of its various isoforms, and various invalid commercial antibodies against BORIS or its specific isoforms have resulted in incomplete/contradictory findings on the expression pattern of BORIS in cancer cells/tissues. On the other hand, tumors are composed of heterogeneous combination of cells that exhibit distinct phenotypic characteristics and proliferative potentials, with having only a fraction of cells expressing BORIS. Accordingly, the level of BORIS transcript/protein might also depend on the grade of malignancy/benignity of tissues, leading to the detection of various expression levels for BORIS in different samples of the same type of tumor.

## Function of BORIS in cancer cells/tissues

**Fig. 1:**
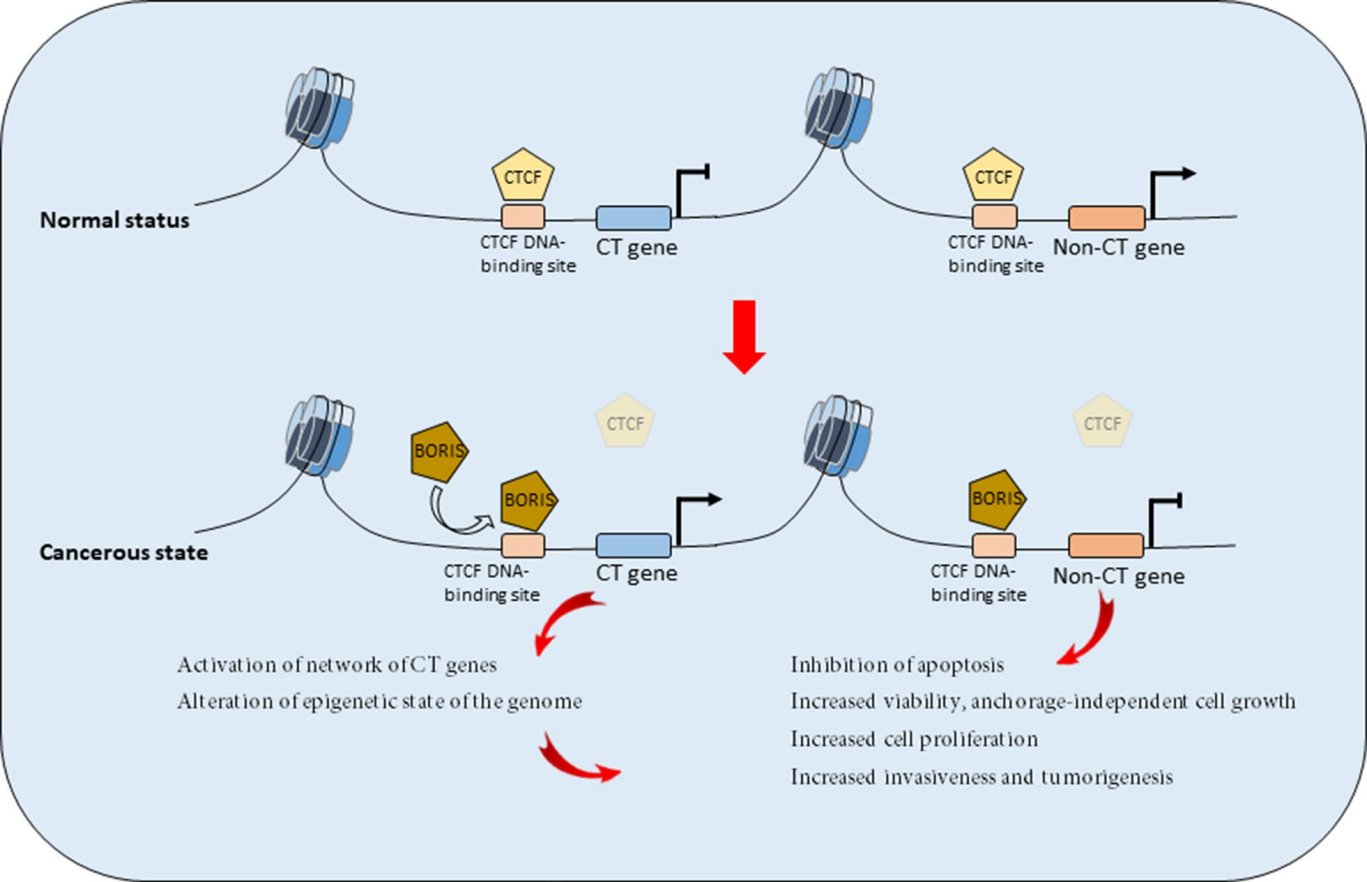
Role of BORIS in tumorigenesis. Increased expression of BORIS, shifts the competition between CTCF and BORIS for binding to CTCF DNA-binding site in favor of BORIS. This leads to the replacement of CTCF by BORIS at promoters of some cancer-testis (CT) genes including MAGE-A1, MAGE-A2, MAGE-A3, MAGE-A4, MAGE-B1, MAGE-B4, GAGE-3-8, RAGE-2, NY-ESO-1 (CTAG1B), LAGE-1 (CTAG2), FerT and TSP50, and some non-CT genes such as BRCA1, Oct-3/4 (POU5F1), MYC, Rb2/p130, SBSN, and hTERT, and androgen, progesterone and estrogen receptors. Expression of target genes leads to cancer progression via activation of the network of CT genes, inhibition of apoptosis, induced cell growth, and increased proliferation and invasiveness of cancer cells

Expression of BORIS in cancer cells likely leads to its interference with CTCF function by competition for binding to CTCF DNA binding target sites. Due to their distinct amino- and carboxy-termini and different interacting proteins, the two proteins of BORIS and CTCF have opposite effects on gene expression [1]. While CTCF represses gene expression and blocks cell proliferation by arresting cells in a senescence-like state throughout the cell cycle [18, 62], BORIS associates with relatively open chromatin of active genes, and appears to activate a unique class of genes like oncogenes and CT genes (Fig. 1). Furthermore, the enforced expression of BORIS in fibroblasts leads to a significant decrease in apoptosis induction, increased anchorage-independent cell growth, and extended lifespan [7, 11, 18, 21, 25, 26, 34]. In contrast, the down-regulation of BORIS with specific siRNAs results in decreased cell proliferation/viability and induced cell death/apoptosis [49, 63] (Fig. 1).

**Fig. 2:**
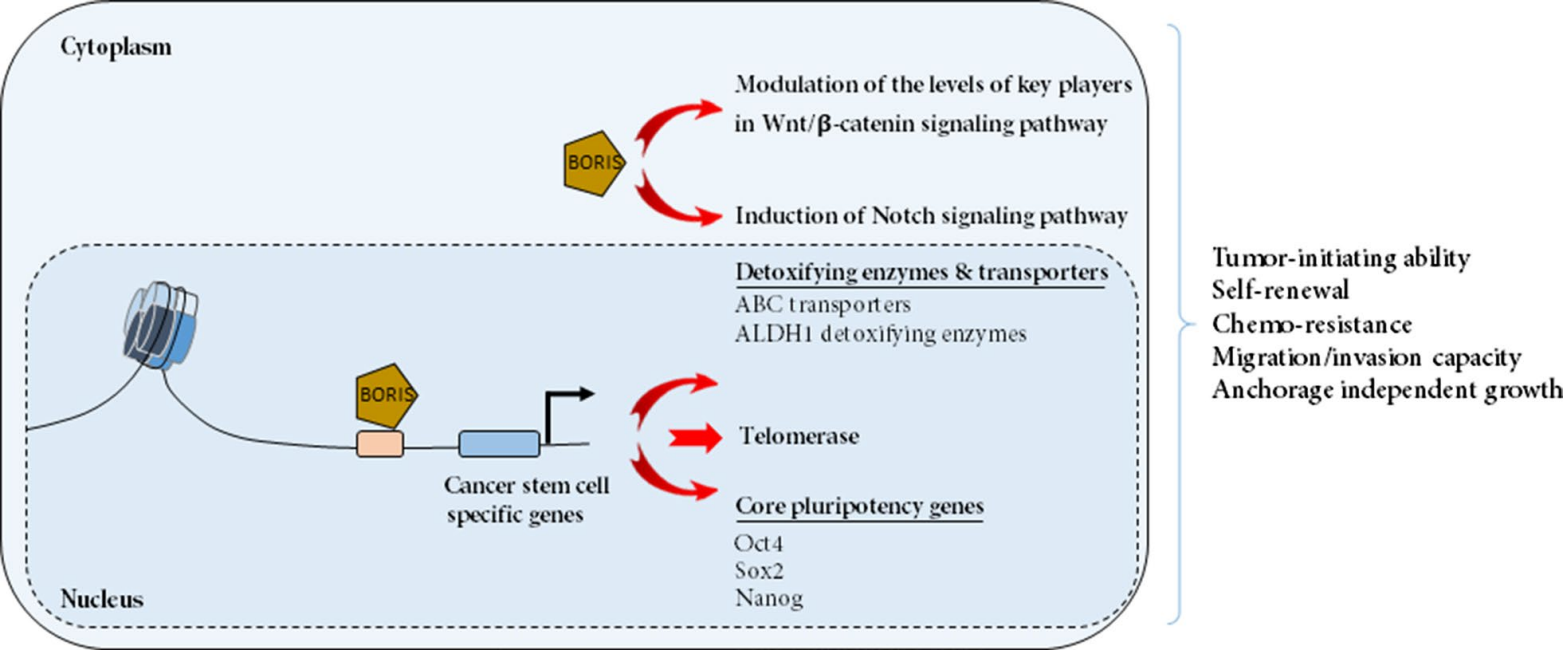
BORIS function in sustaining cancer stem cell (CSC) properties. BORIS induces the expression of some important CSC markers such as ALDH1, ABCG2, hTERT, NANOG, OCT4 and SOX2 in cancer cells. BORIS is also recognized as an inducer of Wnt and Notch signaling pathways that play important roles in the maintenance of CSC properties such as self-renewal, tumor-sphere formation, chemoresistance, anchorage independent growth, and migration/invasion capacity

The results of some studies seem to support this theory that the ectopic expression of BORIS in normal human fibroblasts or low expressing cell lines would induce the replacement of CTCF by BORIS at promoters of several CT genes including MAGE-A1, MAGE-A2, MAGE-A3, MAGE-A4, MAGE-B1, MAGE-B4, GAGE-3-8, RAGE-2, NY-ESO-1 (CTAG1B), LAGE-1 (CTAG2), FerT and TSP50, resulting in the de-repression of the target CT genes [11, 18, 25, 26, 29, 64–67]. In addition to CT genes, BORIS participates in regulation of some non-CT genes such as BRCA1, Oct-3/4 (POU5F1), MYC, Rb2/p130, SBSN and hTERT (human telomerase reverse transcriptase) which are known to be involved in cancer progression [30, 34, 68, 69] (Figs. 1, 2).

Maintenance of telomeres is necessary to inhibit replicative senescence. Telomerase activity which is required to stabilize telomere length has not been detected in differentiated somatic cells, but is detected in proliferative immortal cells, such as germ cells, stem cells, and cancer cells [70–72]. In the majority of telomerase-positive cells such as cancer cell lines and tumors, hypermethylation of hTERT exon 1 region prevents the binding and prevents the repressive effects of CTCF [73–76]. Although methylation of exon 1 region is the most prevalent mechanism to regulate hTERT in tumor cells and tissues, it is found that in some cancer cells, the expression of BORIS prevents the repressive effects of CTCF on hTERT gene, and permits its transcription [34]. Therefore, the expression of BORIS could be an alternative mechanism for the induction of hTERT in cancer cells. This indicates that BORIS might have important regulatory roles in tumor immortalization during tumorigenesis. In breast tumors, estrogen and progesterone have been demonstrated to promote tumorigenesis [35, 36]. It is accepted that BORIS activates the promoters of genes for progesterone and estrogen receptors, suggesting a role for BORIS in the progression of breast tumors [15]. In a similar role, in prostate cancer, androgen receptor (AR) mediates various functions of androgens essential for cell viability, development and invasion in both androgen dependent and independent prostate cancers [37, 38]. BORIS is capable to activate the expression of endogenous AR gene in prostate cell lines. This indicates that BORIS might be involved in the growth and proliferation of prostate tumors [48]. Taken together, these results show the involvement of BORIS in tumorigenesis, cell proliferation and invasiveness of cancer cells and could point to an oncogenic role for BORIS in cancer (Fig. 1).

On the other hand, there are also opposite viewpoints on the role of BORIS in cancer. Several findings discuss that BORIS is not a leading CT gene and its presence is not necessary for the expression of CT genes [11, 16, 25, 66, 77–79]. For example; it has been shown that the expression of some MAGE-A family and of other CT genes in melanoma, glioma stem cells and head and neck squamous cell carcinoma (HNSCC) is observed in the absence of BORIS, suggesting that BORIS might not be an obligatory factor for the activation of CT genes. Accordingly, BORIS positive tumors do not necessarily express high levels of other CT genes and the exogenous expression of BORIS does not always lead to the hypomethylation of promoter in CT genes [16, 27, 64, 78, 80]. Furthermore, in contrast to the previous findings that show opposing roles for BORIS and CTCF, in one study it has been detected that BORIS similar to CTCF caused a significant reduction in cell proliferation and clonogenic capacity. Thus, against previous hypothesis that considers an oncogenic property for BORIS, these data indicate that BORIS and CTCF might act as a tumor suppressor. Accordingly, expression of BORIS in many cancers implies that genetic and epigenetic dysregulation in cancer might result in BORIS induction. Therefore, BORIS can be an effect rather than a cause of transformation [6, 7].

Opposite results in the overexpression of BORIS in different cell types might be a cell context-dependent phenomenon. For example, it is possible that along with BORIS, other transcription or epigenomic regulatory factors be effective to induce CT gene expression and these factors may be expressed in specific cell types. It is also likely that a particular isoform of BORIS is necessary for the regulation of some CT genes. Furthermore, some of the differential effects of BORIS may be attributable to a dose-dependent effect of BORIS on activation of downstream targets and the number of CTCF sites occupied by BORIS. For instance, Gaykalova et al. showed that only lower BORIS concentrations stimulate the highest expression of suprabasin gene as a non-CT target of BORIS, while higher concentrations of BORIS has less inducer effects [79].

In conclusion, according to some reports, BORIS is recognized as a main participator in the induction of some important CT and non-CT genes in cancer, and thus has a role in growth, proliferation, invasion and tumorigenesis of cancer cells. However, the epigenetic state of the cell, the level of expression of genes in CT gene network, and the level of expression of BORIS itself may affect proliferation and tumorigenesis of cancer cells (Fig. 1).

## BORIS in cancer stem cells

In addition to cancer cells, BORIS expression has been detected in some pluripotent cells including human embryonic stem (ES) [13, 44] and embryonal carcinoma (EC) cells (TERA-1, TERA-2, NT2 and NCCIT) [30, 43]. Pluripotent cells and undifferentiated tumor cells share several hallmark traits including self-renewal and differentiation ability, which provide the basis of unlimited proliferative capacity, immortality, and capacity to produce progenitors that differentiate into other cell types [81–84]. Furthermore, expression of some important pluripotent markers including OCT4, SOX2, KIF4 and c-MYC genes which are essential for the maintenance of pluripotency, have been found in many cancer cells and tumors [85–96], indicating that transformation to a cancerous state requires some characteristics found in stem cells [97]. As a result, BORIS might be involved in the establishment of a state of pluripotency, which is also present in a subpopulation of cancer cells called cancer stem cells (CSCs). This hypothesis has been proved to be true by detection of BORIS expression in cancer stem cells (CSCs) and identification of its role in the induction of some important CSC markers and maintenance of CSC properties (Fig. 2).

Cancer stem cells are a group of pluripotent cells that have been detected in most types of solid and hematologic cancers. Similar to normal stem cells, CSCs have uncontrolled proliferation ability, enhanced potential to self-renew, and differentiation capacity into non-CSC progeny [83, 98, 99]. Moreover CSCs, have a higher intrinsic resistance to conventional therapies, such as chemotherapy and radiotherapy through a variety of mechanisms such as increased expression of detoxifying ALDH enzymes, enhanced DNA repair activity, reduced drug activation via quiescence, and increased drug efflux by up regulation of ATP-binding cassette (ABC) transporters [100–104]. Indeed, enhanced activation of one or more signal transduction pathways including the Notch, Hedgehog (HH), and Wnt pathways has been observed in CSCs of many different cancer types. These pathways play an important role in the maintenance of self-renewal potential and ability to avoid being affected by chemo and radio therapy in CSCs [83, 105–112] (Fig. 2). Accordingly, this sub-population of tumor cells plays an important role in tumor growth, recurrence, metastasis, and resistance to treatment. There are various methods for the identification and isolation of CSCs including cell sorting by fluorescence-activated cell sorting (FACS) or magnetic activated cell sorting (MACS) based on the expression of specific surface biomarkers [113–115], sphere-forming assay which is an in vitro method to evaluate the ability of CSCs to form spheres in serum-free medium by anchorage independent growth in suspension [116–119], and finally, functional cell sorting based on biological characteristics of the cells (side population (SP) cell sorting). SP phenotype is a CSC property that defines the cells that express ABC drug transporters, such as MDR1 (P-glycoprotein) and ABCG2. SP cells are characterized by the efflux of fluorescence dyes such as Hoechst 33342 and Rhodamin 123 [120–122].

There is some evidence that support a potential relationship between BORIS expression and CSCs. Expression of BORIS has been found in some EC  [30, 43], and CSCs from neuroblastoma, cervical, colon and breast cancers [39–42]. In addition, expression of BORIS shows a positive correlation with specific stemness and CSC markers [40, 43, 44]. Thus, BORIS is considered to be a positive regulator of cancer/stem cell markers, and to have a role in the maintenance of CSC population in tumors [34, 39, 40, 44].

Related to the presence of BORIS in CSC-enriched population of cancer cells, BORIS mRNA was detected at significantly higher levels in SP cells and tumor spheres compared to non-SP and parental cells [39]. Furthermore, Garikapati et al. showed that CD44/CD133 positive cells that are recognized as CSCs in neuroblastoma have higher levels of BORIS in comparison with CD44/CD133 negative cells [40]. In other studies, BORIS was found to be expressed in cervical and colon CSCs [41, 42]. These findings are in accordance with the findings of Yamada and colleagues indicating that considerable numbers of CT genes including BORIS are expressed in cancer stem like cells. They classified these CT genes in a sub-category called cancer/testis/stem (CTS) genes which define a class of genes expressed in the testis and CSCs [42]. In another research by Pugacheva el al. it was observed that BORIS expression in hES cells disappears upon differentiation, indicating an association with pluripotency [13].

In addition to detecting BORIS in pluripotent and CSCs population, a mutual relationship was found between the expression of BORIS and some fundamental CSC markers and traits. As a noteworthy example, BORIS positive cells express ABCG2 and do not take up Hoechst, so are defined as SP cells [39]. More investigations showed higher level of stem cell (NANOG, OCT4, SOX2) and cancer stem cell (CD44 and ALDH1) markers in BORIS-positive cells in comparition to BORIS-negative cancer cells [40, 43]. In hepatocellular carcinoma tissues, correlation of BORIS expression with liver CSC marker CD90 is another reason for its correlation with CSC markers [46]. Association of BORIS and CSC markers were reinforced when it was observed that some CSC markers such as ALDH1, NANOG, OCT4, SOX2 and ABCG2 were generally down-regulated in all tumor cells after BORIS silencing. In addition, overexpression of BORIS also significantly increased the expression of previously mentioned CSC markers [39–42, 123]. As it was previously implied, one of the cancer/stem cell markers that is inducible by BORIS is hTERT telomerase gene [34]. It is shown that the expression of telomerase is essential for self-renewal of CSCs [124–126]. Therefore, BORIS might be an important factor in self-renewal and immortal capacity of CSCs by induction of hTERT. BORIS has also been recognized as an essential factor for maintaining CSC properties. For example, correlation of BORIS with sphere formation, tumor-initiating ability and maintenance of CSCs in cervical, colon, and breast cancer has been shown in separate reports [39, 41]. Moreover, Garikapati et al. Showed that the expression of BORIS effectively controlled tumurosphere formation and anchorage independent growth in neuroblastoma CSCs [40]. A recent study has reported that BORIS affects the CSC-like traits of human liver cancer cells such as self-renewal, tumor sphere-forming ability, tumorigenicity, chemo resistance and migration/invasion capacity through regulating of OCT4 gene expression [123]. The POU domain transcription factor OCT4 by regulating target genes such as NANOG and SOX2 has been recognized as the most important pluripotency factor and master regulator in the maintenance of CSC-like phenotypes such as self-renewal, chemo-resistance migration and invasion [127–130]. Consequently, in accordance with these findings, BORIS may serve as a biomarker of CSCs and has a probable role in sustaining the stemness properties of CSCs (Fig. 2).

In a research by Soltanian et al. retinoic acid induced differentiation of P19 (as a pluripotent embryonal carcinoma cell line), was concomitant by significant depression of some important pluripotency markers such as OCT4, NANOG and SSEA1, and was not accompanied with significant variations of BORIS expression. In fact, P19 cell line is a heterogeneous population of cells comprising a small population of BORIS-expressing cancer stem cells. Therefore, in order to investigate the changes in the expression level of BORIS during retinoic acid induced differentiation of CSCs, stem-like cells must be first isolated according to their markers and properties [39, 40].

## Association of BORIS with advanced stages of cancers

According to CSC hypothesis, CSCs typically represent a small proportion of total cells of a given tumor that involve in tumor growth, recurrence, metastasis, and treatment resistance. Therefore, it has been shown that CSCs are more frequent in highly aggressive and refractory tumors [118, 131]. Applying this hypothesis to many studies that highlighted the correlation of BORIS expression with poor overall survival of different cancer patients/poorer differentiation grade and recurrence of cancer emphasize that BORIS has a decisive role in maintaining CSCs.

Furthermore, function of BORIS as an inducer of CSC markers and CSC-like traits is consistent with a lot of reports that show expression of BORIS to be associated with poor overall survival/more severe malignancy and advanced stages in different cancers [14]. For instance, in epithelial ovarian cancer and cervical cancer, high level of BORIS is associated with poorer prognosis/less median survival times of patients and advanced cancer stages [41, 45]. BORIS expression was also correlated with tumor size, differentiation grade and tumor recurrences in hepatocellular carcinoma. In this kind of cancer, patients with high BORIS expression had reduced overall survival rate which suggests that BORIS could be used as a diagnostic index of liver cancer [46, 47]. In prostate cancer a positive correlation has also been detected between higher levels of BORIS and higher Gleason score (which measures prostate tumor differentiation and predicts the aggressiveness of the disease), higher T-stage (which reflects the progression of the cancer, e.g., tumor size, metastatic potential, invasiveness), and increased androgen receptor protein levels [48]. Moreover, in prostate cancer, greater BORIS/CTCF ratio was detected in cancer and metastases compared to benign tissue, and an increase in this ratio correlated with higher Gleason’s grade, positive surgical margins, and increased tumor volume [132]. In another report, BORIS expression was significantly associated with lymph node metastasis in esophageal squamous cell cancer (ESCC), and patients with BORIS-positive tumors had a poor overall survival in this cancer, suggesting that BORIS is associated with metastatic activity of ESCC cells in the early stage and BORIS can be considered as a potential biomarker for esophageal cancer patients with a poor prognosis [49]. A research on endometrial cancer showed that increased BORIS mRNA expression level associates with cancer progression and poor survival, so that all the clinically established markers for aggressive endometrial carcinoma including high age, non-endometroid histology, high grade, and hormone receptor loss were significantly associated with high BORIS mRNA levels [32]. In addition, Schwarzenbach el al. indicated that serum levels of cell-free BORIS mRNA were significantly higher in patients with invasive carcinomas than in patients with benign breast diseases or healthy women [133]. Another study on laryngeal squamous cell carcinomas revealed that patients having BORIS 7+ (BORIS transcript variants containing exon 7)/BORIS 7− (BORIS transcript variants lacking exon 7) ratio ≥ 1 had a higher rate of disease relapse than patients with BORIS 7+/BORIS 7− ratio < 1 [50].

## BORIS and its mechanistic connections to tumorigenesis

The mechanism of BORIS function in regulating cancer stemness as well as tumorigenesis has been shown by its involvement in modulating two important signaling pathways in CSCs including Wnt/β-catenin and Notch (Fig. 2). It has been confirmed that abnormal Wnt/β-catenin signaling pathway plays an important role in the maintenance of CSC properties and epithelial–mesenchymal transition (EMT) in various cancers [134–139]. EMT is a process by which epithelial cells gain migratory and invasive properties to acquire features similar to mesenchymal stem cells. This process plays a critical role in cancer metastasis [140]. It is proved that EMT produces CSC like cells with self-renewal and migratory capability [96, 141, 142]. In this regard, it has been shown that BORIS can modulate the levels of Wnt5a, β-catenin, TCF, and pLRP as key players of Wnt/β-catenin signaling pathway. Hence, by regulation of metastasis/EMT through Wnt/β-catenin pathway, BORIS is responsible for cancer stemness [40]. A relationship between BORIS and EMT phenotype has also been confirmed in BCM1 cells as micrometastatic breast cancer cells gathered from bone marrow of breast cancer patients. Interestingly, these cells which express high level of BORIS have some cancer stem cell characteristics and EMT like invasive phenotype [133, 143, 144]. Additionally, BORIS has also been recognized as an inducer of Notch pathway and its expression has been detected in cell lines derived from several solid tumors overexpressing NOTCH3 [110, 145–149]. All together, these results indicate that BORIS plays a principle role in the maintenance of cancer stemness by interacting with WNT/ß-catenin and Notch signaling pathway.

**Fig. 3:**
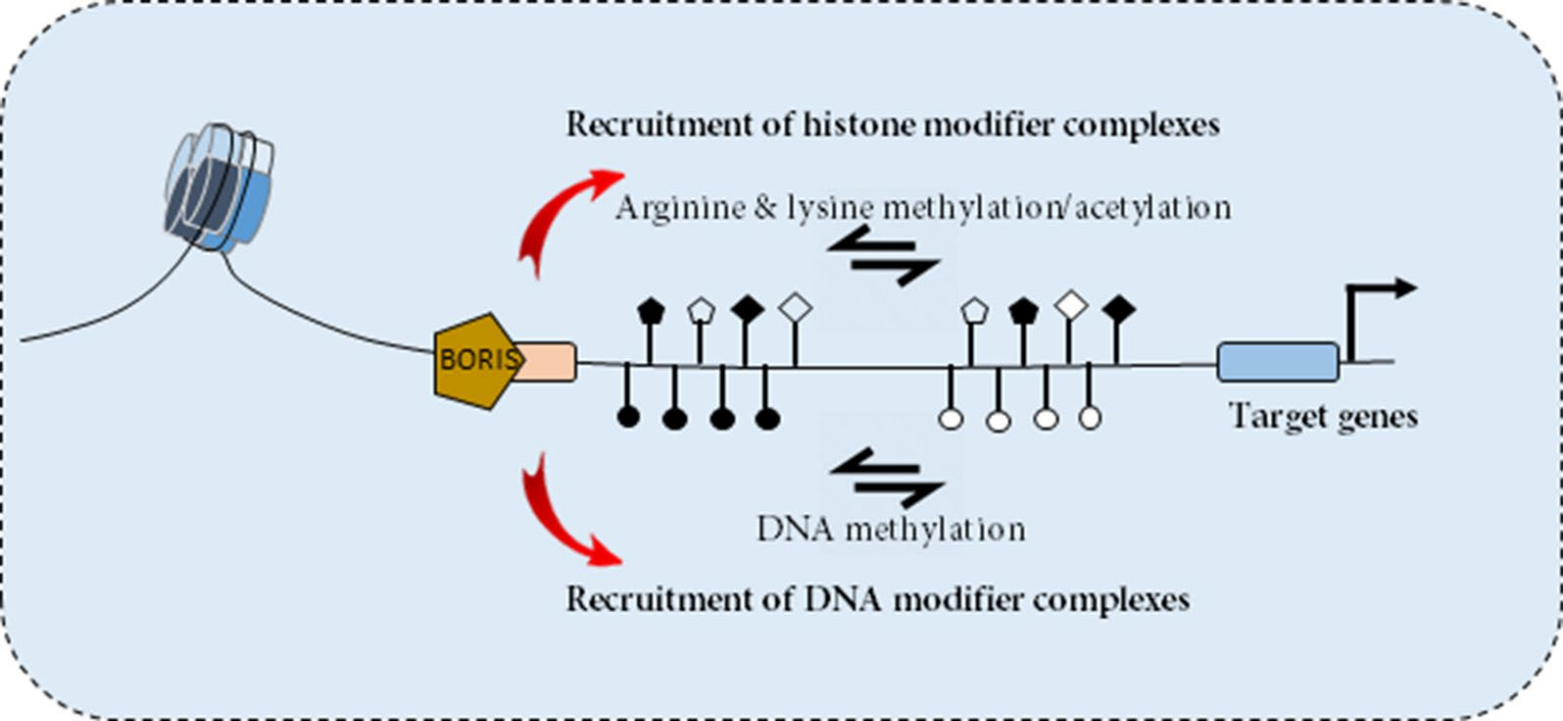
Role of BORIS as an epigenetic modifier regulating the expression of multiple proto-oncogenes and CT genes. BORIS recruits histone modifier complexes to the promoter of target genes to induce tri-methylation of lysine 4 of histone H3 tail, and methylation of arginine and acetylation of lysine 14 of histone H3 tail. BORIS also is involved in promoter methylation/demethylation of target genes. Therefore, BORIS creates a permissive/active chromatin conformation via epigenetic modifications that lead to the upregulation of target genes such as CT genes, Oct4 and Notch3

It has been reported that BORIS is an epigenetic modifier, and its binding to promoters of target genes leads to the recruitment of additional transcription factors or chromatin-remodeling proteins that alter the epigenetic status, chromatin conformation, and transcription of these genes (Fig. 3). For example, it has been demonstrated that *BORIS/CTCF* expression ratio is associated with DNA hypomethylation [45]. Moreover, the overexpression of BORIS is correlated with aberrant expression of multiple proto-oncogenes and CT genes such as NY-ESO-1, MAGE-A1 and MAGE-A3 via induction of promoter demethylation [11, 25, 26, 69, 79, 150]. Furthermore, in vitro studies show recruitment of PRMT7 and SET1A to chromatin induced by BORIS [12]. SET1A and PRMT7 have been recognized as a H3K4 methyltransferase and arginine methyltransferase, respectively [68, 151]. Therefore, BORIS induces the expression of MAGEA1-A4, BAG1, BRCA1, SBSN, NY-ESO-1 and MYC genes via recruitment of histone modifiers onto the promoters of target genes which results in permissive/active histone modifications such as trimethylation of lysine 4 of histone H3 tail (H3K4me3) and acetylation of lysine 14 of histone H3 tail (H3K14Ac) [25, 66, 68, 79, 152, 153]. The role of BORIS as a chromatin regulator protein was confirmed by its ability to bind in NOTCH3 promoter and increasing the H3K4me3/H3K27me3 ratio leading to abnormal upregulation of NOTCH3 in cancer cells [145]. A recent study by Liu el al. showed that BORIS promotes CSC-like traits of human liver cancer cells by epigenetic up-regulating of OCT4. In fact, BORIS regulates OCT4 gene expression via histone methylation modification as reflected by increasing the H3K4me2/H3K27me3 ratio and creates a permissive/active chromatin conformation [123] (Fig. 3). Another mechanism for BORIS-mediated activation of genes has been reported for NY-ESO-1. Kang et al. provided evidence that recruitment of Sp1 to NY-ESO-1 promoter is a mechanism by which BORIS induces NY-ESO-1 in lung cancer [29]. Sp1 is a transcription factor that activates promoters via recruitment of additional regulatory proteins, leading to the formation of a functional transcriptional machinery [154, 155]. Altogether, the results of these studies suggest that BORIS acts as a type of genomic recruiter/chromatin scaffold that recruits many interacting partners and induces open chromatin conformation in the promoter of target genes. This conclusion was further supported by the finding that BORIS is co localized with H3K4me3 and Pol II at transcriptionally active promoters [21].

## Conclusion

In this review we describe the expression pattern and functions of BORIS or CTCF-like protein which has been identified as a paralog of CTCF, an old protein with known functions and pattern of expression. Although there are contradictory reports on the expression pattern and function of BORIS, but it has been recognized as a CT gene that is normally expressed in male germ line cells in testis, and is frequently deregulated in many cancers. In cancer cells, BORIS appears to regulate the activation of other CT genes and oncogenes, affecting cell proliferation and invasive ability of cancer cells. Recent reports show a correlation between BORIS and CSCs. According to these finding, BORIS has also been recognized as an inducer of some important CSC markers and as a probable player in the maintenance of CSCs in advanced cancers. However, further studies are needed to clarify the role of BORIS in sustaining CSC properties, and in advanced stage/poorer differentiation grade cancers.

## Abbreviations

CT: cancer/testis
CSCs: cancer stem cells
BORIS: Brother Of the Regulator of Imprinted Sites
CTCFL: CCCTC-binding factor like
ICR: imprinting control region
hTERT: human telomerase reverse transcriptase
HNSCC: head and neck squamous cell carcinoma
EC: embryonal carcinoma
ABC: ATP-binding cassette
FACS: fluorescence-activated cell sorting
MACS: magnetic activated cell sorting
SP: side population
CTS: cancer/testis/stem
ESCC: esophageal squamous cell cancer
EMT: epithelial–mesenchymal transition
